# Complete genome sequence of the goatpox virus vaccine isolate LRIGP01

**DOI:** 10.1128/mra.01495-25

**Published:** 2026-06-18

**Authors:** Zesmin Akter, Khandaker Sharmin Ferdous, Fouzia Jafrin, Sajedul Hayat, Rokshana Parvin, Md. Zahangir Hosain, Md. Mostofa Kamal

**Affiliations:** 1 Livestock Research Institute, Mohakhali, Dhaka, Bangladesh; 2 Department of Microbiology and Hygiene, Bangladesh Agricultural University 54492, Mymensingh, Bangladesh; 3 Department of Pathology, Bangladesh Agricultural University 54492, Mymensingh, Bangladesh; Katholieke Universiteit Leuven, Leuven, Belgium

**Keywords:** goatpox, vaccine, WGS, Bangladesh

## Abstract

We present the complete genome sequence of the goatpox virus vaccine isolate LRIGP01 from the Livestock Research Institute in Bangladesh. The genome is 150,342 nucleotides long and encodes 151 genes with a 25.3% GC content. Phylogenetically, the isolate clusters with several goatpox isolates from India.

## ANNOUNCEMENT

Goatpox is a highly contagious viral disease of goats caused by the goatpox virus (GTPV), belonging to the *Capripoxvirus* goatpox species in the *Poxviridae* family (1). The disease spreads through direct contact, fomites, and respiratory droplets (2). There is no specific treatment, but vaccines and biosecurity measures can prevent outbreaks (3). We report the complete genome of the goatpox virus vaccine isolate LRIGP01 from the Livestock Research Institute in Bangladesh.

The virus was isolated from infected goat lung tissue in 1992 (4) and was propagated and attenuated through passage 44 in Vero cell culture. The live-attenuated virus from passage 44 served as the master seed. One milliliter of reconstituted seed was inoculated onto semi-confluent Vero cells and maintained in MEM with 2% FBS, 2% L-glutamine, and 1% antibiotic-antimycotic at 37°C in 5% CO_2_ and harvested at 70%–80% CPE. Viral DNA was extracted from the culture fluid using the QIAamp DNA Mini Kit (QIAGEN, Germany). Library construction employed the Rapid Plus DNA Lib Prep Kit for Illumina (ABclonal, USA), involving fragmentation with a Covaris LE220R-plus (Covaris, USA), end polishing, A-tailing, and ligation with Illumina adapters, followed by PCR. PCR products were purified with 1.0× AMPure XP (Beckman Coulter, USA), quality assessed on an Agilent 5400 (Agilent, USA), and quantified by real-time PCR. The qualified library was sequenced on the Illumina NovaSeq 6000 (Illumina, USA) using a PE150 strategy at Novogene (China), yielding 17,231,226 paired-end reads with Phred scores above Q34.

The genome was assembled through a multi-stage pipeline. The initial genome assembly was performed on Galaxy Europe (5). Raw FASTQ reads were quality-trimmed with Fastp v1.0.1 (6), mapped to reference genome GP5-Indora (PV167794.1) using Bowtie2 v2.5.4 (7) to extract paired reads for *de novo* assembly with SPAdes v4.1.0 (8), resulting in five contigs with minimum and maximum lengths of 10,813–80,999 bp. The assembly was scaffolded with RagTag v2.1.0 (9). To finalize the sequence, this draft assembly was refined using Geneious Prime v2025.1.3 (10) with default settings. Raw fastq reads were imported as paired-end sets, quality-trimmed with BBDuk v38.84 (11), and mapped to the draft assembly using the GP5-Indora genome as a scaffold by Geneious mapper to bridge the five initial contigs, resolve terminal regions, verify accuracy, and fill remaining gaps. This iterative process produced a final 150,342 bp consensus sequence with a GC content of 25.3% and 13.9× coverage, which was submitted to GenBank. The sequence was aligned with goatpox genomes in GenBank using MAFFT v7.490 (12), and their percentage similarity was determined. Potential open reading frames were identified using the best match option in Annotate from the database in Geneious Prime.

**Fig 1:**
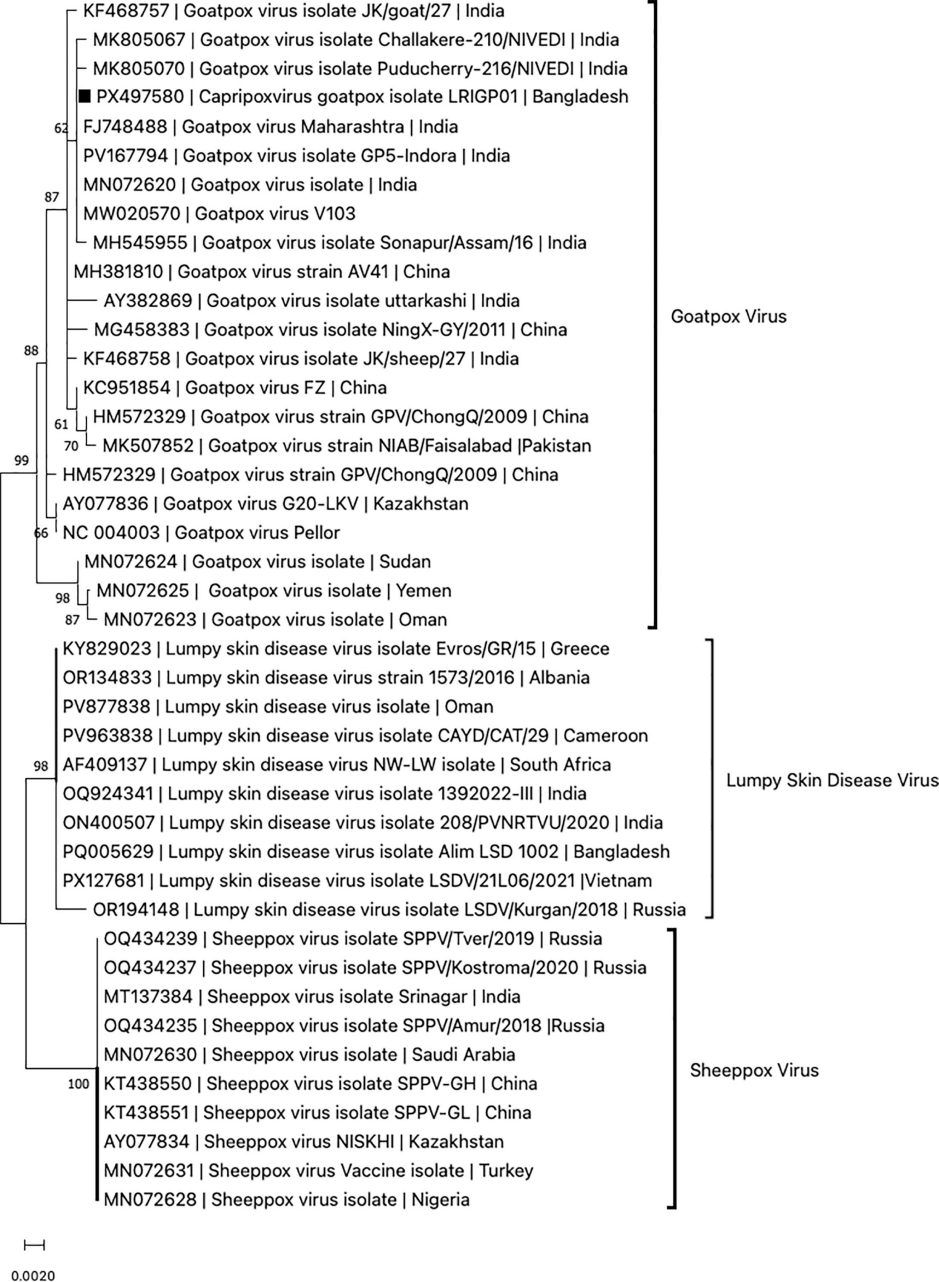
Phylogenetic analysis of the goatpox virus LRIGP01 was performed in MEGA 11 (14) using full-length nucleotide sequences of the representative *Capripoxvirus* P32 gene retrieved from the NCBI database. Sequences were aligned using the MUSCLE algorithm. A maximum likelihood tree was constructed using the Tamura 3-parameter (15) model, selected based on the highest log-likelihood value (−1,534.01), with 1,000 bootstrap replicates.

In BLAST analysis (13), LRIGP01 exhibited 98.55%–99.89% similarity to other goatpox genomes. The highest was 99.89% with GTPV strain FZ (KC951854.1), 99.31% with NCBI reference strain goatpox virus Pellor (NC_004003.1), and up to 97.60% with other capripoxviruses. The genome encodes 151 genes and both left and right inverted terminal repeats of ~2.4 kb and concatemer resolution sites. In the phylogenetic tree (Fig. 1), the isolate LRIGP01 clustered with several goatpox isolates from India.

## Data Availability

The complete genome sequence of the goatpox virus vaccine isolate LRIGP01 has been submitted to the NCBI GenBank database under BioProject number PRJNA1328371, BioSample number SAMN52653508, SRA number SRR35777226, and accession number PX497580.

## References

[1] Hamdi J, Bamouh Z, Jazouli M, Alhyane M, Safini N, Omari Tadlaoui K, Fassi Fihri O, El Harrak M. 2021. Experimental infection of indigenous North African goats with goatpox virus. Acta Vet Scand 63:9. doi:10.1186/s13028-021-00574-2 33663573 PMC7931584

[2] Hopker A, Pandey N, Saikia D, Goswami J, Hopker S, Saikia R, Sargison N. 2019. Spread and impact of goat pox (“sagolay bohonta”) in a village smallholder community around Kaziranga National Park, Assam, India. Trop Anim Health Prod 51:819–829. doi:10.1007/s11250-018-1759-4 30649668 PMC6469614

[3] Bukar BA, Mabu IM. 2023. Common diseases of goats, treatment and preventive measuresGoat science - from keeping to precision production. IntechOpen.

[4] Islam MR, Kamaruddin KM. 2007. Laboratory manual for homologus goatpox vaccine production 1st ed. Bangladesh Livestock Research Institute.

[5] Community TG. 2024. The galaxy platform for accessible, reproducible, and collaborative data analyses: 2024 update. Nucleic Acids Res 52:W83–W94. doi: 10.1093/nar/gkae410 38769056 10.1093/nar/gkae410 PMC11223835

[6] Chen S, Zhou Y, Chen Y, Gu J. 2018. Fastp: an ultra-fast all-in-one fastq. Preprocessor. doi:10.1101/274100 PMC6129281 30423086

[7] Langmead B, Salzberg SL. 2012. Fast gapped-read alignment with Bowtie 2. Nat Methods 9:357–359. doi:10.1038/nmeth.1923 22388286 PMC3322381

[8] Bankevich A, Nurk S, Antipov D, Gurevich AA, Dvorkin M, Kulikov AS, Lesin VM, Nikolenko SI, Pham S, Prjibelski AD, Pyshkin AV, Sirotkin AV, Vyahhi N, Tesler G, Alekseyev MA, Pevzner PA. 2012. SPAdes: a new genome assembly algorithm and its applications to single-cell sequencing. J Comput Biol 19:455–477. doi:10.1089/cmb.2012.0021 22506599 PMC3342519

[9] Alonge M, Soyk S, Ramakrishnan S, Wang X, Goodwin S, Sedlazeck FJ, Lippman ZB, Schatz MC. 2019. Ragoo: fast and accurate reference-guided scaffolding of draft genomes. Genome Biol 20:224. doi:10.1186/s13059-019-1829-6 31661016 PMC6816165

[10] Geneious Prime. 2025. http://www.geneious.com.

[11] Bushnell B, Rood J, Singer E. 2017. Bbmerge - accurate paired shotgun read merging via overlap. PLoS One 12:e0185056. doi:10.1371/journal.pone.0185056 29073143 PMC5657622

[12] Katoh K, Standley DM. 2013. Mafft multiple sequence alignment software version 7: improvements in performance and usability. Mol Biol Evol 30:772–780. doi:10.1093/molbev/mst010 23329690 PMC3603318

[13] Altschul SF, Gish W, Miller W, Myers EW, Lipman DJ. 1990. Basic local alignment search tool. J Mol Biol 215:403–410. doi:10.1016/S0022-2836(05)80360-2 2231712

[14] Tamura K, Stecher G, Kumar S. 2021. Mega11: molecular evolutionary genetics analysis version 11. Mol Biol Evol 38:3022–3027. doi:10.1093/molbev/msab120 33892491 PMC8233496

[15] Tamura K. 1992. Estimation of the number of nucleotide substitutions when there are strong transition-transversion and G+C-content biases. Mol Biol Evol 9:678–687. doi:10.1093/oxfordjournals.molbev.a040752 1630306

